# Effects of Program Representation on Pointer Analyses — An Empirical Study

**DOI:** 10.1007/978-3-030-71500-7_12

**Published:** 2021-02-24

**Authors:** Jyoti Prakash, Abhishek Tiwari, Christian Hammer

**Affiliations:** 8 Universidad Autónoma de Madrid, Madrid, Spain; 9 University of Twente, Enschede, The Netherlands; 10 University of Potsdam, Potsdam, Germany; 11 National University of Singapore, Singapore, Singapore

**Keywords:** Pointer Analysis, Java, Program Analysis, Empirical Studies

## Abstract

Static analysis frameworks, such as *Soot* and *Wala*, are used by researchers to prototype and compare program analyses. These frameworks vary on heap abstraction, modeling library classes, and underlying intermediate program representation (IR). Often, these variations pose a threat to the validity of the results as the implications of comparing the same analysis implementation in different frameworks are still unexplored. Earlier studies have focused on the precision, soundness, and recall of the algorithms implemented in these frameworks; however, little to no work has been done to evaluate the effects of program representation. In this work, we fill this gap and study the impact of program representation on pointer analysis. Unfortunately, existing metrics are insufficient for such a comparison due to their inability to isolate each aspect of the program representation. Therefore, we define two novel metrics that measure these analyses’ precision after isolating the influence of class-hierarchy and intermediate representation. Our results establish that the minor differences in the class hierarchy and IR do not impact program analysis significantly. Besides, they reveal the sources of unsoundness that aid researchers in developing program analysis.

## 1 Introduction

Researchers have proposed various approaches to enhance the precision and soundness of static analyses [6, 9, 10, 14, 17, 26, 30, 31]. They use program analysis frameworks to prototype and evaluate their algorithms. A program analysis based on declarative specifications (a growingly popular implementation paradigm) uses these frameworks to extract fundamental dataflow relations and feeds them as the ground facts to a Datalog engine.

Program analysis frameworks, primarily Soot and Wala, are being increasingly adopted in program analysis [11, 31, 40]. These frameworks provide APIs, which abstract internal program representation. However, program representation in these frameworks is heterogeneous in many aspects. A few of those are:

- *Intermediate Representation (IR).* The intermediate language for program representation is an abstraction of the object code (bytecode) or source code. It removes syntactic sugar from the language and transforms it into a (minimal) core language. Thus, analysis developers can focus on the core language features to define their analysis.
- *Modeling of libraries in analysis scope.* Real-life applications are seldomly developed from scratch; instead, they reuse library modules. Whole-program analyses consider these libraries for soundness in terms of the class-hierarchy, which forms the analyses' scope. Users can tune the scope to favor scalability over soundness.
- *Heap Modeling.* Heap modeling is the technique to model dynamic heap allocation statically. Precise heap modeling is undecidable; therefore, analyses use approximations to keep it decidable [20]. Apart from these approximations, optimization may choose to keep a low memory footprint at the cost of precision and soundness.

These factors influence the precision, scalability, soundness of the analyses, and at the same time, impede a fair comparison of analyses. Earlier research (Späth et al. [29]) was concerned about the validity of results when comparing two analyses frameworks. Reif et al. consider the comparison of different frameworks "bogus" [21] at the outset. Although many earlier works have proposed techniques to enhance scalability and precision, little to no work was done on how program representation influences program analyses. As a result, a comparison of new analyses with existing analyses suffers from a threat to validity that might have been overlooked. In this work, we fill the gap with an empirical study of these aspects of program analysis frameworks.

We choose pointer analysis for this study. Pointer analysis computes the heap locations referred by program variables and builds the foundation for many others, such as alias analysis, type-state, or program slicing. To evaluate intermediate representation and library modeling, we choose Doop, a *state-of-the-art* pointer analysis framework and compare its analysis for different frontends. For the third aspect, heap modeling, we compare the pointer analysis of *Wala*'s (a *state-of-the-art* program analysis) framework with *Doop* using *Wala*'s frontend, i.e., leveraging the identical intermediate representation.

A challenging aspect of this work is that the existing notions of precision for pointer analysis were insufficient. The computation of these metrics does not isolate single aspects of pointer analysis but rather combines all effects. For example, the average points-to set size is influenced by all three of the aforementioned aspects. It is difficult to determine the effect of each aspect by only looking at the score. In this work, we counteract this problem by introducing metrics that isolate a particular aspect under study and nullifies the effect of others. Therefore, we define two novel metrics in section 3.1, one for measuring the effects of libraries to enable a fair comparison among frameworks. To the best of our knowledge, it is the first study that evaluates the impact of program representation on pointer analysis. Precisely, in this paper, we make the following contributions:

- We defined two metrics for evaluating each aspect in isolation, one for modeling of library classes, the other for IR.
- We evaluated the differences in library modeling and found that these have little influence on program analyses. Additionally, we discovered sources of unsoundness in these frameworks.
- We evaluated the precision for different IRs and found that they have no impact on the precision of *virtual* method call elimination.
- We empirically found differences in heap abstractions even for analyses claiming the same levels of context-sensitivity regarding the types of heap objects.

In summary, our empirical study dispels the threats to the validity of the results of existing works posed by these differences of frameworks. It also discovers novel sources of unsoundness and imprecision in existing frameworks that provide suggestions that users/developers of these frameworks could incorporate into their analyses. Although we focus on pointer analysis in the paper, our results are, in principle, generalizable to many other static analyses, as the findings presented in this paper also hold for these. We have made the artifacts available on https://github.com/jpksh90/pointeval to facilitate reproduction.

## 2 Background and Motivation

The goal of pointer analysis is to determine which objects a variable may refer (point) to at runtime. A *points-to set* is a static approximation of this question, which maps variables to objects that are allocated on the heap (heap objects). More precisely, if V is the set of variables in a program, and H is the set of heap objects, then $\textit{points-to} : \mathrm{V} \rightarrow \mathcal{P}(\mathrm{H})$. $\textit{points-to}(v)$ returns the set of heap objects in H referred by $v$.

Doop is a framework that exclusively focuses on pointer analysis, defines the analysis' inference rules in Datalog [41], and is in active development. It supports tuning of the analysis to adapt for various factors of precision (and scalability). Doop leverages the program synthesizer Soufflé [12, 22] to resolve *points-to* according to the inference rules and the ground facts, which are derived directly from the program.

Wala [37] and Soot [28] are general-purpose program analyzers providing some pre-defined analyses and APIs for the development of custom analyses. Wala comes with various pre-defined pointer analyses [39], some of which feature novel optimizations to enhance scalability.

A *context-sensitive analysis* improves a pointer analysis' precision by discerning method calls based on their calling contexts. Popular notions of contexts are based on method callsites [23] (*callsite-sensitive*), invoking objects (*object-sensitive*) [19], or hybrids thereof [13].

In the sequel, we explain the need for this study by exemplifying the three factors that influence the results of pointer analyses.

Listing 1.1: Factory Method

```
1 public class Factory {
2     public static void main(String args[]) {
3       AInt a = AInt.getInstance(5);
4       AInt b = AInt.getInstance(7); } }
5 class AInt {
6     private Integer a; //... getter, setter and constructor
7     public static AInt getInstance(int x) {
8             return new AInt(x); //allocation a@8
9 }}
```

Listing 1.2: Soot IR for the *main* method in Listing 1.1

```
1 public class Factory extends java.lang.Object {
2     //constructor
3   public static void main(java.lang.String[]) {
4       java.lang.String[] r0;
5         AInt r1, r2;
6       r0 := @parameter0: java.lang.String[];
7       r1 = staticinvoke <AInt: AInt getInstance(int)>(5);
8       r2 = staticinvoke <AInt: AInt getInstance(int)>(7);
9         return; } }
```

## 2.1 Intermediate Representation

Many program analyses tools leverage an *intermediate representation* (IR) instead of the actual source or bytecode for analysis. IRs remove syntactic sugar from the source code and make it amenable to analysis by focussing on the fundamental operations. Popular strategies for IR generation are based on three-address code or *static single assignment* (SSA) form [4]. By default, the Soot framework uses a three-address-based IR (*Jimple*) [35], while Wala uses a SSA-based IR [38]. Both IRs are register-based [36,38], and hence introduce synthetic variables to mimic the stack-based Java bytecode. *Doop* can be configured to leverage either *Jimple* or *Wala*'s IR as a frontend for program representation.

Consider the code example in Listing 1.1 and its Jimple IR depicted in Listing 1.2. The *main* method declaration (line 2) translates to the almost identical line 3 in the IR, whose parameter is translated to the variable *@parameter0* (line 6). Due to the additional local variable *r0* (line 4), the single main method argument translates to two variables in the IR. The invocations of the static method *getInstance* (lines 3 and 4 of Listing 1.1) are translated to the corresponding operation code *staticinvoke* with the method name and arguments. The newly allocated objects returned from these factory method invocations are stored in the variables *r1* and *r2*.

Wala's IR generation differs significantly from Soot (see Listing 1.3). As an SSA-based IR, it does not assign names to method parameters and variables but ordinal numbers (starting from '*1*') called *variable numbers* (we prepend '*v*' to these numbers for clarity). Thus, the receiver object (*this* reference in Java), or the first parameter in the case of a static method is (silently) assigned

Listing 1.3: Wala IR for the *main* method in Listing 1.1

```
Factory.main([Ljava/lang/String;)V
5 = invokestatic < Application, LAInt,
    getInstance(I)LAInt; > 3 @1 exception:4
8 = invokestatic < Application, LAInt,
    getInstance(I)LAInt; > 6 @7 exception:7
return
```

Listing 1.4: Snapshot of pointer analysis results from Doop with different IR

```
1 // Variables in main method with ****Wala****
2 < <<main method array>> <Factory: void
    main(java.lang.String[])>/v1
3 // Variables in main method with ****Soot****
4 > <<main method array>>    <Factory: void
    main(java.lang.String[])>/@parameter0
5 > <<main method array>>    <Factory: void
    main(java.lang.String[])>/l0#_0
```

the number *v1*. Further method parameters are assigned subsequent variable numbers, succeeded by local variables. Again, the static method calls to the method *getInstance* are translated to *invokestatic*, where *v3* and *v6* hold the (implicitly defined) constant arguments 6 and 7. The objects returned from the factory method invocations are stored in the variables *v5* and *v8*. Potential exceptions thrown in the invoked methods are stored in *v4* or *v7*, respectively.

The differences in program representation influence the metrics of pointer analysis: We analyzed Listing 1.1 context-insensitively with Doop, using Jimple and Wala's IR. The results are shown in Listing 1.4: The main method parameter object `«main method array»` is referred by one variable in Wala (line 2) but two variables in Soot (lines 4– 5). Even though the average points-to set size is 1 for all variables in Listing 1.4, we found noticeable differences in the average points-to set sizes in other program's analyses, with Soot's frontend the average size of the points-to set being 2.07 for 3328 variables, and 1.95 for 2298 variables using Wala's—Jimple again created more variables than Wala. These subtle differences in program representation affect the average points-to set size, and it is unclear whether these two numbers are in fact comparable. In this work, we aim to investigate the impact of IRs on the precision and scalability of the analysis (Section 4.3).

## 2.2 Static modeling of libraries

As a whole program analysis, a pointer analysis does not only requires knowledge of the program to be analyzed but also the library classes, especially those related to the runtime. For example, a whole program analysis of a Java application would require the runtime libraries, such as those in *rt.jar*, and other dependent libraries, bundled with the application. Analysis frameworks such as Soot and Wala construct the class hierarchy based on all classes present in libraries and the application. They can also remove "irrelevant" classes, favoring scalability over soundness. Interestingly, we found cases where some frontends do not load all of the required classes, which induces discrepancies when comparing the analyses.

Consider the program shown in Listing 1.1. To corroborate our intuition, we analyzed this program context-insensitively with Soot's and Wala's front-ends. Using the former front-end, Doop loads 3,837 classes and computes the analysis with an average points-to set size of 2.07. With Wala's front-end, it loads 19,927 (~5×) classes for analysis with an average points-to set size of 1.95. Further investigating the types of heap objects, we found that Doop with Wala's IR contains objects of the class *java.security.PrivilegedActionException*, which is absent in the analysis with Soot. Note that our simple program contains no instance of that type, so it must stem from analyzing libraries. In another instance, Soot loads the classes from *javax.crypto*, whereas Wala does not. In this research, we examine the imprecise modeling and discover possible implications on precision and soundness (sections 4.1 and 4.2).

### 2.3 Heap Abstraction

Heap abstraction is an important aspect of pointer analysis and determines how object allocations are statically represented in the analysis. One simple approach is to create a unique representation for each object allocation site in the program (*allocation site abstraction*). However, at runtime allocation sites can be executed more than once, creating several objects that are then represented by the same abstract value. As an example, consider the object allocation (line 8) of Listing 1.1, represented via a single abstract object, say *a@8*. In the *main* method the newly allocated objects returned by *getInstance* are captured by the variables *a* and *b*, which would both refer to the abstract object, *a@8* in the result of the pointer analysis. Thus, *a* and *b* are spuriously considered *aliases* (i.e., refering to the same object.) This imprecision stems from ignoring the calling-context of *getInstance* (*context-insensitive heap abstraction*).

A *context-sensitive heap abstraction* (a.k.a *heap cloning*) discerns the abstract[3] heap-objects based on the calling context, associating the calling context with the heap object to distinguish the allocations in a pair ⟨*allocation site*, *call stack*⟩. Thus the allocation at line 8 is represented as two heap objects, ⟨*a@8*, 3⟩ and ⟨*a@8*, 4⟩. Without loss of generality, the length of the call stack can be increased to any finite number, lest the analysis be undecidable. All *state-of-the-art* pointer analysis frameworks offer *context-sensitive heap abstraction* with a finite context length.

The discussion above demonstrates how the choice of heap abstraction can (potentially) influence pointer analysis. Therefore, in this work, we study the frameworks' heap abstractions. We conducted a preliminary study to gain initial insights and to validate our intuition, and context-sensitively analyzed Listing 1.1 with a *one-call-site context-sensitivity* in Doop with Wala's IR, and the *one-call-site sensitive* analysis of the Wala framework. Both of these analyses

[3] In the sequel we will reference abstract heap objects as heap objects for brevity.

use a context-sensitive heap abstraction with context length of one. In spite of that, Wala creates 17 objects while Doop creates 133 objects (~7×). The average points-to set size varies between 1.55 for the analysis provided by Wala and 1.62 for Doop with Wala's IR[4]. Thus, we can see that even with the same level of sensitivity in heap abstraction (and IR), analysis results depend on the framework used. Manual inspection revealed that Wala selectively uses the context-sensitive heap abstraction, applying contextual heap abstraction only to non-library classes while treating the library's objects context-insensitively. Out of the 17 heap objects, Wala uses context-sensitivity for only 6 objects. In contrast, Doop leverages context-sensitivity for all heap objects, including the library's objects. These initial insights motivated us to analyze the influence of heap abstraction on precision and scalability in more detail in Section 4.4.

To summarize, the parameters for program analysis such as IR (Section 2.1), static modeling of libraries (Section 2.2), and heap abstraction (Section 2.3) affect the precision and scalability of a pointer analysis. Based on initial insights, we analyze the influence of the mentioned parameters using different frameworks, frontends, and on a larger and diverse set of benchmark applications.

## 3 Methodology

### 3.1 Metrics Used

The precision of a pointer analysis has been defined in numerous ways in the literature. Some of the metrics for precision available in the literature are the average size of the points-to sets, the number of call-graph edges, and the number of resolved virtual calls. These metrics are not clearly superior to one another but rather tailored to specific clients, for example, the latter is leveraged by compilers in *devirtualization* of virtual method calls.

All of these metrics reflect how precisely the analysis computes the points-to sets (sets of heap objects referred by a variable). For example, whether or not a virtual call can be resolved depends on the heap objects' types in the points-to set of the target variable. If there is only one type (or subtypes thereof that do not redefine the virtual method) then the virtual call is resolvable. Therefore, the precision of a client analysis depends on how precisely the points-to set for each variable in the program can be resolved, in other words, how low the value of the average points-to set size is. An average size close to one is considered the hallmark of pointer analysis [27].

Therefore, we leverage the wide-spread metric of average points-to set size for our evaluation, i.e., the ratio of the total sizes of the points-to sets to the total number of local variables [26,34]. It permits a client-agnostic comparison of the pointer analysis, which generalizes our evaluation results to any specific analysis. We refer to the average points-to set size as *precision* in this paper. Note that the actual precision of the analysis is inversely connected to the average points-to

[4] Note that due to context-sensitive analysis, the average points-to set size is better than that mentioned in sections 2.2 and 2.1.

set size: A lower precision value (i.e. average points-to set size) implies a higher precision of the computed analysis result, as precise analyses aim at excluding unrealizable (at runtime) allocation sites from the points-to sets of variables.

An IR may create many synthetic variables, among other reasons for method parameters or for $\phi$-nodes at control-flow joins of SSA-form. For example, three-address code re-uses the same variable in assignments in the *if* and *else* blocks of a conditional. However, SSA-based IRs insert a synthetic variable in a $\phi$-node at the control-flow join to select one of the distinct variables of the respective blocks. The presence of synthetic variables in IRs impedes the comparison of different analyses using the average points-to set size, as averages depend on the (unequal) number of variables. Therefore, we devise heuristics to establish comparability of our metrics for different IRs.

Another challenge in this work is inferring the impact of each analysis parameter on its precision. Computed at the end of the analysis, the average points-to set size loses information on the contribution of a particular aspect of pointer analysis. Therefore, we require a fine-grained metric to quantify the precision for each parameter. We propose two such techniques, one for the class hierarchy and the other for the intermediate representation.

**Class Hierarchy** The analysis of the program's class hierarchy builds the foundation for inferring relevant variables and heap allocations. However, each framework leverages a particular strategy to infer classes that contribute to the program's semantics. Adding irrelevant classes to the class hierarchy may manifest into a synthetically precise analysis, as these classes add to the total number of variables (which will all be pointing to an empty set), thus potentially decreasing the average size of points-to sets. Some of these variables and heap allocations are not part of the actual code executed at runtime, but rather arise out of an imperfect model of the program analysis framework's frontend. Here, we study the variables and heap objects stemming from the additional classes exclusive to a framework.

We first instrument the *Doop* framework to log the class hierarchies and compare the class hierarchies obtained using Soot and Wala as frontends, which yields the classes exclusive to each of the frameworks. $CH_{soot}$ and $CH_{wala}$ denotes the set of classes in the class hierarchies of Soot and Wala respectively. $CH_{common} = CH_{soot} \cap CH_{wala}$ is the set of classes common to both frameworks. We define *CH-precision* in terms of the average points-to set size restricted to variables defined in methods of $CH_{common}$.

**Definition 1.** CH-Precision *(CP). Let* $\mathrm{V}_f^c$ *be the set of variables defined in methods of* $CH_{common}$ *for the frontend* $f \in \{soot, wala\}$*, and* $\mathrm{H}_f^c(v) = \{h \mid h \in points\text{-}to(v), v \in \mathrm{V}_f^c\}$*. CH-Precision is the ratio of* $\mathrm{H}_f^c$ *and* $\mathrm{V}_f^c$*, i.e.,*

$$CP_f = \frac{\sum\limits_{v \in \mathrm{V}_f^c} \mathrm{H}_f^c(v)}{|\mathrm{V}_f^c|}$$

If an analysis does not contain any exclusive classes or all of their variables (and corresponding heap objects) belong to the types present in the set of exclusive classes, CH-precision equals the average points-to set size.

**Intermediate Representation (IR)** The choice of IR determines a program's representation but retains the program's semantics, particularly with respect to heap allocations. Thus, different IR's can differ in the number of variables but will not introduce additional heap objects (e.g. Listing 1.4).A fundamental difference between Soot's Jimple and Wala's SSA-based IR is that SSA creates unique variables for each variable definition, while three-address code does not. Rendering our precision metric comparable for structurally different IRs is challenging, as tracking which variables correspond to each other is technically involved and may not be unique. Therefore, we rely on a heuristic to determine comparable variables. We motivate the heuristics considering two different IRs for the *main* method in Listing 1.1. Jimple (Listing 1.2) defines four variables, `r0` – `r2`, and `parameter0`, while Wala's IR (Listing 1.3) defines three variables: `v1` (implicit, not shown in the listing), `v5`, `v8`.

**Definition 2.** *Defm denotes the set of variables defined in a method.* $\mathit{Defm}(m, ir) = \bigcup_{s_i \in S_{m,ir}} \mathit{def}(s_i)$*, where* $S_{m,ir}$ *is the set of statements in method* $m$ *for* $ir$*,* $\mathit{def}(s_i)$ *the variables defined in* $s_i$*.*

**Definition 3.** Interesting Method. *A method* $m$ *is interesting if* $|\mathit{Defm}(m, wala)| \neq |\mathit{Defm}(m, jimple)|$ *and* $m$ *is defined in class* $C \in CH_{common}$*, i.e., the number of variables defined in the method with the same signature vary for different IRs.* $\mathcal{M}$ *denotes the set of interesting methods.*

To determine the set of interesting methods ($\mathcal{M}$) we leverage the logs from pointer analyses and segregate the variables in the logs according to the declaring method ($m$). If the sizes of the corresponding sets differ for a method $m$, it is considered interesting. ($\mathcal{M}$ is confined to the set of methods defined in $CH_{common}$ to exclude the exclusive classes.) Subsequently, we determine the points-to relation for the variables in $\mathcal{M}$.

Simple average of the heap objects and number of variables is insufficient for comparing the precision of the analysis between two IRs. Differences in class hierarchies and aliasing generates new variables, which makes the ratio incomparable if the heap objects are not same. To circumvent this problem, we combine average points-to set size with ideas from virtual call resolution. The number of virtual call sites in a program is identical irrespective of the differences in program representation (caused by aliasing and redundant variables). Therefore, we receive a fair comparison if we restrict the average point-to set size to the target variables of virtual method calls. We define a new metric, *average devirtualized heap objects* ($H_v^f$), which is the ratio of the total size of points-to sets of target variables at the virtual call sites to the number of virtual call sites.

**Definition 4.** Average devirtualized heap objects *(*$H_v^f$*). For the set of virtual call-sites* $C$ *in the IR of a framework* $f$ *and* $V_{C,f}$ *as the set of invoking variables*

*at C, let* $H_v$ = *points-to*$(v)$ *be the set of heap objects referred by* $v \in V_{C,f}$. *Average devirtualized heap objects is*

$$H_v^f = \frac{\sum_{v \in V_{C,f}} points\text{-}to(v)}{|C|}$$

Based on the above discussion, we formulate and answer the following research questions:
**RQ1.** How does the class hierarchy vary with the benchmarks?
**RQ2.** How do differences in class hierarchies affect the precision of analyses?
**RQ3.** How do the choice of IR affect the precision of the analysis?
**RQ4.** How do the heap abstractions differ between pointer analysis frameworks?

## 4 Evaluation

We use Doop version *4.20.7-67* and *Wala* version *1.5.0*. For RQ1-RQ3, we invoked Doop with the following analysis options: `1-call-site-sensitive`, `1-object-sensitive`, `2-call-site-sensitive+heap`, `2-object-sensitive+heap`. Specific options used in our study for each research questions are described in their respective sections. We use the DaCapo [2] (version 9.12-bach) benchmarks, a standardized suite of open-source Java applications, for our study.

### 4.1 RQ1: Class hierarchy differences with benchmarks

We captured the class hierarchies considered by the analyses to determine the differences. We instrumented *Doop* to log the classes considered during a (context-insensitive) analysis, which yields the complete class hierarchy. In order to investigate whether the class hierarchy depends on the frontend, we performed this experiment with Soot and Wala as frontend[5]. Table 1 lists the differences in the class hierarchies using Soot and Wala. On an average, Wala exclusively contains ~13,994 classes in its class hierarchy. The number of classes exclusive to Wala range from 12,524 (Xalan) to 16,707 (Tradebeans). Soot's class hierarchy on average contains 26 classes not present in Wala's, ranging from zero to 62.

In the case of PMD and H3, Soot's class hierarchy contains only a single additional class, Jython has an additional 2 classes. Eclipse, Lusearch, and Luindex contain 62, 53, 53 additional classes, respectively. In the remaining cases the class hierarchy in Soot is strictly a subset of Wala's. In next RQ, we will study the impact of these additional classes on the precision and scalability of the analysis.

### 4.2 RQ2: Precision differences with class hierarchy

[5] Note that Soot and Wala provide options to exclude certain classes from analysis (to, e.g., exclude library classes). For a fair comparison we ignore this feature and compute the whole class hierarchy including libraries.

Table 1: Difference in classes considered by Soot and Wala. Last two columns show the extra classes loaded by Soot and Wala respectively.

| | #classes analyzed | | Extra classes | |
|---|---|---|---|---|
| Benchmark | Wala | Soot | Soot | Wala |
| Avrora | 21,997 | 9,204 | 0 | 12,793 |
| Batik | 23,461 | 10,739 | 12 | 12,734 |
| Eclipse | 25,718 | 9,813 | 62 | 15,967 |
| H2 | 21,007 | 8,042 | 1 | 12,966 |
| Jython | 23,323 | 10,411 | 2 | 12,914 |
| Lusearch | 20,469 | 4,671 | 53 | 15,851 |
| Luindex | 20,479 | 4,681 | 53 | 15,851 |
| PMD | 21,315 | 8,517 | 1 | 12,799 |
| SunFlow | 20,677 | 7,847 | 0 | 12,830 |
| Tradebeans | 20,658 | 3,951 | 0 | 16,707 |
| Xalan | 22,688 | 10,164 | 0 | 12,524 |

**Study Setup** We have used the *var-points-to* relation, which maps all variables and context pairs to their resolved pairs of heap-object and context. We select those variables that originate from classes common to both frameworks (Section 4.1) and query their points-to information. We then compute the $CH-Precision$ based on Definition 1.

**Results** Table 2 presents the results of the analysis (for one-callsite, one-object, and two-object context-sensitivity) for the objects and variables belonging to exclusive classes present in Wala (only non-zero values included). Note that the two-object sensitive analysis did not terminate for Eclipse and Jython, therefore, these are not presented in the table. In one-callsite and one-objects analysis, Table 2 lists six out of eleven benchmarks contain variables that belong to the exclusive class hierarchy. The remaining benchmark applications show no differences in the number of variables and heap-objects, despite the presence of additional classes. It demonstrates that the additional classes loaded by the these frameworks have no influence on the precision of these benchmarks.

The third and fourth columns of Table 2 list the number of variables (in principle, variable-context pairs) and heap objects belonging to the set of exclusive classes, respectively. In all analyses, all but one benchmark have a higher average points-to set size for exclusive variables than the general average. Tradebeans only creates 3 additional heap objects with Wala' frontend, therefore the analyses are almost identical for both frontends. The average points-to sets for exclusive classes for bigger benchmarks such as Eclipse and Jython are outliers, showing very high averages. Still, the contribution of exclusive classes' heap objects and variables is negligible compared with the total heap objects of these benchmarks.

The eighth and ninth columns depict the CH-precision and the original precision for the analyses. We observe that the CH-precision is slightly lower than the precision for all benchmarks but tradebeans, which originates from the addi-

Table 2: Differences in precision in the presence of additional objects in class hierarchy (Wala). HO denotes the sum of number of heap objects in *points-to* set. $CP_{wala}$ is the precision score for variables in $CH_{common}$.

| | | Exclusive classes | | | Original | | | |
|---|---|---|---|---|---|---|---|---|
| Analysis | Benchmark | Vars. | HO | Average | Vars. | HO | Precision | $CP_{wala}$ |
| *1CS* | avrora | 19 | 297 | 15.63 | 96,680 | 883,798 | 9.141 | 9.140 |
| | eclipse | 453 | 171,071 | 377.64 | 1,231,854 | 61,556,548 | 49.970 | 49.850 |
| | h2 | 31 | 321 | 10.35 | 78,154 | 639,202 | 8.178 | 8.177 |
| | jython | 35 | 17,682 | 505.2 | 289,244 | 8,000,917 | 27.661 | 27.603 |
| | tradebeans | 3 | 3 | 1 | 59,853 | 549,391 | 9.179 | 9.179 |
| | xalan | 39 | 2,466 | 63.23 | 147,488 | 1,911,750 | 12.962 | 12.948 |
| *1OS* | avrora | 19 | 14,844 | 781.26 | 82,972 | 404,231 | 4.871 | 4.694 |
| | eclipse | 388 | 329,008 | 847.95 | 1,053,618 | 46,337,474 | 43.979 | 43.683 |
| | h2 | 31 | 2747 | 88.61 | 59,800 | 220,058 | 3.679 | 3.635 |
| | jython | 35 | 147,214 | 4,206.11 | 573,823 | 22,152,008 | 38.604 | 38.35 |
| | tradebeans | 3 | 4 | 1.33 | 45,807 | 154,883 | 3.381 | 3.381 |
| | xalan | 39 | 13,831 | 354.64 | 199,404 | 1,576,762 | 7.907 | 7.839 |
| *2OS* | avrora | 19 | 1752 | 92.21 | 119,805 | 348,368 | 2.907 | 2.893 |
| | h2 | 31 | 1195 | 38.54 | 82,795 | 242,667 | 2.930 | 2.917 |
| | tradebeans | 3 | 4 | 1.33 | 57,200 | 197,808 | 3.458 | 3.458 |
| | xalan | 55 | 4268 | 77.6 | 362,885 | 1,733,576 | 4.777 | 4.766 |

Table 3: Differences in precision in the presence of additional objects in class hierarchy for Eclipse (Soot).

| | | Variables | Heap Objects | $CP_{soot}$ | Original |
|---|---|---|---|---|---|
| *1-call-site* | Exclusive Classes | 786 | 3331 | 44.95 | - |
| | Original | 1.5M | 68.5M | - | 44.92 |
| *1-object* | Exclusive Classes | 1020 | 4130 | 44.90 | - |
| | Original | 1.3M | 60.8M | - | 44.87 |

tional heap objects and variables. These primarily belong to the internal libraries such as *sun.util, sun.util.resources* (discussed later).

With the Soot frontend (Table 3), the *CH-Precision* differs from *Precision* only for the benchmark Eclipse, for the other benchmarks the analysis does not contain any objects where the type belongs to the exclusive classes of the frontend. However, it is difficult to compare the precision of Soot v/s Wala on CH-Precision score due to differing variable numbers for the same benchmark application.

> ***Finding 1***: *Differences in class-hierarchy negligibly impact the pointer analysis precision (and thus client analyses).*

*Soundness* In our observation, the Wala frontend takes the internal Java libraries into account. We find heap objects belonging to libraries such as *sun.nio.fs*, *sun.util.resources*, *sun.security*, and *sun.nio.cs*, which are internal libraries used by the JVM. Soot, on the other hand, does not model these libraries for analysis.

Comparing the class hierarchies of the analyses using Soot and Wala, we observed that the class hierarchy using Soot as frontend is a subset of Wala's for all

Table 4: Total (for each framework) and interesting (section 4.3) methods $\mathcal{M}$.

| Benchmark | 1-CS | | | 1-OS | | | 2-OS | | |
|---|---|---|---|---|---|---|---|---|---|
| | Soot | Wala | $\mathcal{M}$ | Soot | Wala | $\mathcal{M}$ | Soot | Wala | $\mathcal{M}$ |
| Avrora | 3651 | 3678 | 3194 | 3642 | 3669 | 3187 | 3615 | 3642 | 3159 |
| Batik | 3407 | 3415 | 3006 | 3398 | 3406 | 2999 | 3285 | 3293 | 2895 |
| Eclipse | 20339 | 20281 | 18723 | 20261 | 20204 | 18655 | Timed out | | |
| H2 | 3041 | 3091 | 2673 | 3027 | 3075 | 2661 | 2985 | 3029 | 2616 |
| Jython | 8482 | 8531 | 7672 | 8447 | 8494 | 7643 | Timed out | | |
| Lusearch | 2449 | 2457 | 2135 | 2440 | 2448 | 2128 | 2414 | 2422 | 2103 |
| Luindex | 3524 | 3532 | 3132 | 3514 | 3522 | 3124 | 3466 | 3474 | 3081 |
| PMD | 4587 | 4596 | 4131 | 4577 | 4586 | 4124 | 4418 | 4427 | 3978 |
| Sunflow | 8369 | 8384 | 7514 | 8335 | 8350 | 7475 | 7740 | 7754 | 6928 |
| Tradebeans | 2442 | 2406 | 2083 | 2433 | 2397 | 2076 | 2407 | 2371 | 2051 |
| Xalan | 4607 | 5701 | 4125 | 4597 | 5678 | 4115 | 4502 | 5503 | 4031 |

benchmarks except Eclipse. This suggests that analyses with Soot are as sound as analyses with Wala for all benchmarks except Eclipse. Eclipse is a compelling case: Its analysis using Soot contains heap objects and variables that belong to the internal libraries of Eclipse, such as *org.eclipse.core.internal.runtime.PerformanceStatsProcessor*, while the analyses with Wala does not report these objects. However, results from the analyses with Wala contain heap objects from the internal libraries such as *sun.util.**, which are not present using Soot. It shows that the class hierarchy model is unsound in both frontends, as both lack some of the classes loaded by these benchmark applications at runtime.

> *Our study reveals that library modeling in both Soot and Wala is* unsound *even for (non-native) Java objects, shown by the presence of heap-objects belonging to the exclusive classes of Soot and Wala.*

### 4.3 RQ3: Precision for IR varies with the framework

**Study Setup** The study setup is similar to Section 4.2. We use the application's var-points-to sets, i.e., the relation of variables and heap objects excluding the library objects. From the results of the three analysis sensitivities, we extract the set of interesting methods ($\mathcal{M}$, Def. 3) and compute the average devirtualized heap objects score for the virtual calls in interesting methods. We use the Jimple IR (`--no-ssa` option in Doop), and Wala's IR (`--wala-fact-gen` option in Doop) for evaluation.

**Results** Table 4 reports the number of interesting methods and total methods resolved using both frontends. Note that the number of interesting method is identical for both frameworks for the same type of context-sensitivity. The number of reachable methods in each analysis differs, just as the number of distinct methods signatures discovered in each framework (columns Soot, Wala in 1-CS, 1-OS, 2-OS[6]). However, deriving a relationship between those is impossible, as

[6] We excluded 2-CS for its large file sizes.

Table 5: Results for IR. Third and fifth columns are the number of heap objects. Fourth and sixth columns are the number of virtual calls. Last two columns lists the average devirtualized heap objects ($H_v^f$) for Soot and Wala respectively.

| | | **Soot** | | **Wala** | | $H_v^f$ | |
|---|---|---|---|---|---|---|---|
| Analysis | Benchmark | Heap Objs. | Virt. Calls | Heap Objs. | Virt. Calls | Soot | Wala |
| *1 call-site sensitive* | Avrora | 7,684 | 3499 | 7759 | 3499 | 2.20 | 2.22 |
| | Batik | 2,645 | 1588 | 2702 | 1588 | 1.67 | 1.70 |
| | Eclipse | 7.7M | 56.8K | 7.9M | 56.8K | 136.33 | 139.24 |
| | H2 | 1,936 | 1,434 | 1,988 | 1,434 | 1.35 | 1.39 |
| | Jython | 662K | 9,286 | 656K | 9,283 | 71.33 | 70.67 |
| | Lusearch | 1,667 | 1,139 | 1,674 | 1,139 | 1.46 | 1.47 |
| | Luindex | 8,090 | 4408 | 8,098 | 4,408 | 1.84 | 1.84 |
| | PMD | 8,518 | 3,527 | 8,708 | 3,527 | 2.42 | 2.47 |
| | Sunflow | 4,741 | 2,088 | 4,627 | 2,088 | 2.27 | 2.22 |
| | Tradebeans | 1,638 | 1,114 | 1,649 | 1,106 | 1.47 | 1.49 |
| | Xalan | 43K | 5,832 | 55K | 5,850 | 7.45 | 9.44 |
| *1 object sensitive* | Avrora | 6,561 | 3,498 | 6,563 | 3,498 | 1.88 | 1.88 |
| | Batik | 1,673 | 1,587 | 1,709 | 1,587 | 1.05 | 1.08 |
| | Eclipse | 2.9M | 56.7K | 3.0M | 56.8K | 51.61 | 53.53 |
| | H2 | 1,218 | 1,433 | 1,258 | 1,433 | 0.85 | 0.88 |
| | Jython | 3.5K | 9,272 | 3.6K | 9,269 | 386.79 | 389.20 |
| | Lusearch | 958 | 1,138 | 964 | 1,138 | 0.84 | 0.85 |
| | Luindex | 4,530 | 4,407 | 4,552 | 4,407 | 1.03 | 1.03 |
| | PMD | 7,369 | 3,527 | 7,518 | 3,527 | 2.09 | 2.13 |
| | Sunflow | 2,978 | 2,088 | 2,864 | 2,088 | 1.43 | 1.37 |
| | Tradebeans | 928 | 1,113 | 938 | 1,105 | 0.83 | 0.85 |
| | Xalan | 99K | 5,830 | 106K | 5,810 | 17.11 | 18.33 |
| *2 object sensitive* | Avrora | 8,561 | 3,459 | 8,563 | 3,459 | 2.47 | 2.48 |
| | Batik | 1,257 | 1,567 | 1,275 | 1,567 | 0.80 | 0.81 |
| | H2 | 1,288 | 1,433 | 1,307 | 1,433 | 0.90 | 0.91 |
| | Luindex | 5,210 | 4,363 | 5,215 | 4,363 | 1.19 | 1.20 |
| | Lusearch | 948 | 1,138 | 954 | 1,138 | 0.83 | 0.84 |
| | PMD | 7,271 | 3,496 | 7,398 | 3,496 | 2.08 | 2.12 |
| | Sunflow | 2,342 | 2,088 | 2,324 | 2,088 | 1.12 | 1.11 |
| | Tradebeans | 919 | 1,113 | 929 | 1,105 | 0.83 | 0.84 |
| | Xalan | 214K | 5,791 | 215K | 5,771 | 36.97 | 37.36 |

analyses such as one-call-site and one-object are not comparable. In all cases, we observed that the majority (~90%) of the methods are interesting. Therefore, we cannot ignore the significance of this aspect.

*Interesting methods are difficult to ignore because of their sheer presence in the benchmarks applications.*

Table 5 presents the differences in the average devirtualized heap objects for Jimple and Wala IR. Although the number of variables and abstract heap locations are dependent on the IR, we did not observe many differences between those when restricting ourselves to target variables of virtual method calls, which corresponds to our intuition. The differences in the $H_v^f$ values for both IRs

Table 6: Differences Soot IR v/s Wala IR for Xalan

| Methods | Wala | Soot | Actual |
|---|---|---|---|
| org.apache.xalan.transformer.TransformerImpl.transformNode() | ✔ | ✖ | ✔ |
| Exceptions | ✔ | ✖ | ✔ |
| org.apache.xalan.xsltc.trax.TransformerFactoryImpl.setFeature() | ✖ | ✔ | ✔ |
| MethodResolver.getConstructor() | ✔ | ✖ | ✔ |
| xerces.xml.dtd.XMLDTDLoader() | ✔ | ✖ | — |
| org.apache.xpath.getSourceTree() | ✔ | ✖ | ✔ |

Listing 1.5: Differences in types of heap objects created in both analysis

```
(Wala) sun.misc.URLClassPath$Loader
(Wala) java.util.zip.ZipError
(Soot) javax.xml.transform.FactoryFinder$ConfigurationError
```

are negligible except for three larger benchmarks, Jython, Eclipse, and Xalan. Overall, the values from Soot IR were smaller than those of Wala, implying that *devirtualization* in Soot is either slightly more precise or slightly less sound than in Wala, however, the differences are minor in the majority of the cases. In conclusion, the choice of IR shows little to no impact on the precision of pointer analysis. In the sequel, we describe one such case study where the difference in $H_v^f$ is approximately two, which is a significant figure as compared to others.

***Finding 2***: *IR has negligible impact on the precision of pointer analysis at least for the devirtualization client.*

*Case Study—Xalan* To further investigate the differences, we chose *Xalan* using a one-call-site analysis as the $H_v^f$ values for Soot (7.45) and Wala (9.44) display the highest difference among all benchmarks. The number of heap objects in both cases differs significantly, with Soot having 43K heap objects, and Wala having 55K heap objects for a comparable number of virtual calls (5,832 vs. 5,850).

To examine the heap objects, we collected their class types. We observed that the types of some of these objects belongs to the classes in $CH_{soot} \setminus CH_{common}$ or $CH_{wala} \setminus CH_{common}$. Listing 1.5 depicts the differences in heap objects created by these frameworks.

We also discovered (potential) sources of imprecision and unsoundness in both analyses. Table 6 lists methods and exceptions missed by both Soot and Wala frameworks. Note that these methods and exceptions belong to the common class hierarchy. We observed that Wala has precise exception modeling compared to Soot. For other virtual methods invocations, we compared the runtime call-graph to the static call-graph. In our observation, both Wala and Soot are unsound, as demonstrated by the absence of certain method calls in the call-graph for both analyses. In addition, Wala imprecisely includes `xerces.xml.dtd.XMLDTDLoader()` into its call-graph (which at least in our experiments was not executed at runtime).

*Apart from reflection, imprecise/unsound virtual call resolution also induces imprecision/unsoundness into the analysis.*

### 4.4 RQ4: Heap abstractions in pointer analysis frameworks

In this section, we compare Doop's analysis using Wala's frontend with Wala's own analysis. We omit the comparison with the Soot framework as it leverages IRs different from Wala's and thus would not be comparable.

Table 7: Number of Heap objects

| Benchmark | #Heap Objects Doop | #Heap Objects Wala | #Types Doop | #Types Wala |
|---|---|---|---|---|
| avrora | 2,504 | 28,235 | 751 | 3,256 |
| batik | 1,699 | 16,724 | 537 | 1,938 |
| h2 | 1,467 | 16,688 | 482 | 1,934 |
| lusearch | 1,242 | 16,274 | 551 | 1,898 |
| luindex | 1,901 | 19,343 | 404 | 2,250 |
| pmd | 2,398 | 31,774 | 734 | 2,498 |
| sunflow | 4,424 | 16,688 | 1,196 | 1,934 |
| tradebeans | 1,230 | 16,734 | 405 | 1,937 |
| xalan | 3,874 | 18,174 | 1,003 | 2,078 |

**Study Setup** We compare the one-call-site sensitive with context-sensitive heap abstraction (unique heap objects for each call-site, heap cloning) analysis available in the Wala framework with a one-call-site with one-level heap abstraction in Doop, and set the time budget to 7 hours. Analyses with a higher level of call-site sensitivity were not scalable in the Wala framework and therefore, we do not leverage those. Other optimizations in Wala, such as the use of object-sensitivity only for collection objects, are not comparable to the object-sensitive analysis available in Doop. Therefore, we also choose to ignore it. To handle reflective calls in Wala, we use the option `REFLECTIONS.FULL`. In what follows, we present the results of our study. We first present the differences in the number of heap objects and, subsequently, delve into its implications.

*Differences in the heap objects* For evaluation, we extracted the heap-objects created in Wala's and Doop's analyses and observe huge differences in the number of heap objects created. Intuitively, using the same level of heap-sensitivity (heap-cloning) should create the same number of heap objects. However, in certain cases, the number of heap objects in Wala exhibits a factor of ~14 compared to those in Doop (columns 2 and 3 in 7). (Note that eclipse and jython are elided, as the analyses did not terminate within the time budget owing to the large file size (~100GB).) Therefore, the heap abstractions of these analyses are not comparable, although superficially they look similar.

*Subtle optimizations also manifests into imprecise heap modeling even though, at the outset, they look similar.*

To investigate this further, we compared the the types of the heap objects. Our study shows that the set of types are not even consistent using the same frontend! In many cases the types of objects analyzed by Wala is approximately four times those in Doop (columns 4 and 5 in Table 7). The differences in heap abstraction for application level objects build the reason for this.

*Application level objects* Application level objects, i.e., the heap objects created due to allocations within the program (rather than libraries.) In three out of eleven benchmarks we observe that Doop's analysis is lacking application level classes that Wala reports. We found corresponding allocations on a manual inspection of the source code. For example, in *avrora*, the analysis in Wala allocates heap objects of *BRNE_builder* [8], which are not present in Doop's. Similar cases can be found in *PMD* and *Xalan*. However, owing to the limitations of the program representation, we could not determine the precise reason for the unsoundness. Pointer analysis uses an IR based on a control flow graph (CFG) rather than source code. Being a lower level representation of the program source code the IR mangles variables names. Therefore, a one-to-one correspondence between the IR's variables and variables in source code is not trivial.

***Finding 3***: *Heap modeling is not similar even for allocations within the application scope. Wala handles application levels objects more precisely than Soot in our evaluation.*

## 5 Threats to Validity

Naturally, the technique used relies on the precise handling of reflection calls and other dynamic features of the languages such as dynamic proxies. Other than that, handling of native calls could alleviate the unsoundness of the analyses. Analysis of native calls could infer the native objects in JVM missed by the Soot framework. Here, we have used the TamiFlex framework for handling reflection calls. Other approaches have improved the reflection handling [10,15–18,25]. To convince ourself, we experimented with one of the *state-of-the-art* techniques, i.e., reflection with matching substring resolution [10]. However, we did not find any significant differences in results. Another limitation of this study is the unsoundness from ignoring the native library calls in static analyses. Few of the sources of unsoundness discovered stem from the native calls. Recently, Fourtounis et al. [7] proposed a technique for resolving native calls in Java. However, at the time of writing this paper, the technique was not available. Further, our analysis in Section 4.3 is based on test-cases which may not reflect all possible executions of an application.

Our study also involves hours of manual evaluation which can be subject to bias. To counteract it, we did a manual inspection of the source code, especially for the sources of unsoundness. We had rerun the benchmark applications with valid inputs to determine to compare and reassert that the objects are actually allocated during runtime.

## 6 Related Work

*Pointer analysis tools* Pointer analysis has garnered significant interest in the last decades, focussing on scalability, precision, and soundness. The Doop system used in this paper results from years of research on declarative-style pointer analysis [1,3,10,24,26]. Similarly, the Wala framework was a result of an industrial project and, unlike Doop, follows an imperative paradigm. The underlying program representation comes with many prior assumptions mentioned. In this work, we study the effects of these assumptions on program analysis.

*Empirical studies on pointer analysis* Recent empirical studies focussed on the soundness limitations from dynamic features of languages in existing pointer analyses and call-graph construction as pointer analysis and call-graph construction are closely related static analyses and are mutually dependent. Dietrich et al. [5] proposed automated and manual techniques to generate unsoundness oracles to test static analysis. Sui et al. [32] present the causes of unsoundness in static analysis frameworks (Soot, Wala, and Doop) due to the dynamic features of languages. Rief et al. [21] did a comprehensive study, focussed on features in Java 9, for call-graph generation algorithms and expose the problems in the *state-of-the-art* esp. related to method calls in the Java runtime. Our work is orthogonal: we evaluate the influence of program representation on program analyses. Here, we rather focus on the program representation in static analysis frameworks and also the unsoundness arising out of it. Our study is also extensible for Java 9.

Sui et al. [33] evaluated the *recall* of call-graph construction and present how it impacts the algorithms in practice. Their evaluation expose the problems in the *state-of-the-art* esp. related to method calls in the Java runtime. Our unsoundness results concur with theirs. Here, we have focussed on program representation rather than the dynamic features of the language, which are hard to analyze for static analyzers. Further, our work features two novel metrics apart from the standard *precision* and *recall*, to measure the impact of different aspects of program representation.

## 7 Conclusion

This paper reports the effects of program representation on program analysis. Our metrics makes it possible to compare implementations leveraging different frontends. We find that differences in program representation have negligible impact on the precision of the pointer analysis. In addition, we also discovered novel sources of unsoundness and imprecision in the program analysis. Our results also demonstrate that the promised heap abstraction are practically not similar, even though they may appear so on a birds eye view. Since pointer analysis builds the foundation of many static analyses, we conjecture the results generalize these, as well.

## References

[1] Antoniadis, T., Triantafyllou, K., Smaragdakis, Y.: Porting doop to soufflé;: A tale of inter-engine portability for datalog-based analyses. In: Proceedings of the 6th ACM SIGPLAN International Workshop on State Of the Art in Program Analysis. pp. 25–30. SOAP 2017, ACM, New York, NY, USA (2017). 10.1145/3088515.3088522, 10.1145/3088515.3088522

[2] Blackburn, S.M., Garner, R., Hoffmann, C., Khang, A.M., McKinley, K.S., Bentzur, R., Diwan, A., Feinberg, D., Frampton, D., Guyer, S.Z., Hirzel, M., Hosking, A., Jump, M., Lee, H., Moss, J.E.B., Phansalkar, A., Stefanović, D., VanDrunen, T., von Dincklage, D., Wiedermann, B.: The dacapo benchmarks: Java benchmarking development and analysis. In: Proceedings of the 21st Annual ACM SIGPLAN Conference on Object-oriented Programming Systems, Languages, and Applications. pp. 169–190. OOPSLA ’06, ACM, New York, NY, USA (2006). 10.1145/1167473.1167488, 10.1145/1167473.1167488

[3] Bravenboer, M., Smaragdakis, Y.: Strictly declarative specification of sophisticated points-to analyses. In: Proceedings of the 24th ACM SIGPLAN Conference on Object Oriented Programming Systems Languages and Applications. pp. 243–262. OOPSLA ’09, ACM, New York, NY, USA (2009). 10.1145/1640089.1640108, 10.1145/1640089.1640108

[4] Cytron, R., Ferrante, J., Rosen, B.K., Wegman, M.N., Zadeck, F.K.: Efficiently computing static single assignment form and the control dependence graph. ACM Trans. Program. Lang. Syst. **13**(4), 451–490 (Oct 1991). 10.1145/115372.115320

[5] Dietrich, J., Sui, L., Rasheed, S., Tahir, A.: On the construction of soundness oracles. In: Proceedings of the 6th ACM SIGPLAN International Workshop on State Of the Art in Program Analysis. pp. 37–42. SOAP 2017, Association for Computing Machinery, New York, NY, USA (2017). 10.1145/3088515.3088520, 10.1145/3088515.3088520

[6] Fourtounis, G., Triantafyllou, L., Smaragdakis, Y.: Identifying java calls in native code via binary scanning. In: Proceedings of the 29th ACM SIGSOFT International Symposium on Software Testing and Analysis. pp. 388–400. ISSTA 2020, Association for Computing Machinery, New York, NY, USA (2020). 10.1145/3395363.3397368, 10.1145/3395363.3397368

[7] Fourtounis, G., Triantafyllou, L., Smaragdakis, Y.: Identifying java calls in native code via binary scanning. In: Proceedings of the 29th ACM SIGSOFT International Symposium on Software Testing and Analysis. pp. 388–400. ISSTA 2020, Association for Computing Machinery, New York, NY, USA (2020). 10.1145/3395363.3397368, 10.1145/3395363.3397368

[8] GitHub: https://github.com/cmorty/. https://github.com/cmorty/avrora/blob/222ea1645b67bc40429881526555d19bced4a590/src/avrora/arch/avr/AVRInstrBuilder.java (August 2020), (Accessed on 05.08.2020)

[9] Grech, N., Fourtounis, G., Francalanza, A., Smaragdakis, Y.: Heaps don’t lie: Countering unsoundness with heap snapshots. Proc. ACM Program. Lang. **1**(OOPSLA) (Oct 2017). 10.1145/3133892, 10.1145/3133892

[10] Grech, N., Kastrinis, G., Smaragdakis, Y.: Efficient Reflection String Analysis via Graph Coloring. In: Millstein, T. (ed.) 32nd European Conference on Object-Oriented Programming (ECOOP 2018). Leibniz International Proceedings in Informatics (LIPIcs), vol. 109, pp. 26:1–26:25. Schloss Dagstuhl-Leibniz-Zentrum fuer Informatik, Dagstuhl, Germany (2018). 10.4230/LIPIcs.ECOOP.2018.26, http://drops.dagstuhl.de/opus/volltexte/2018/9231

[11] Grech, N., Smaragdakis, Y.: P/taint: Unified points-to and taint analysis. Proc. ACM Program. Lang. **1**(OOPSLA), 102:1–102:28 (Oct 2017). 10.1145/3133926, 10.1145/3133926

[12] Jordan, H., Scholz, B., Subotić, P.: Soufflé: On synthesis of program analyzers. In: Chaudhuri, S., Farzan, A. (eds.) Computer Aided Verification. pp. 422–430. Springer International Publishing, Cham (2016), 10.1007/978-3-319-41540-6_23

[13] Kastrinis, G., Smaragdakis, Y.: Hybrid context-sensitivity for points-to analysis. In: Proceedings of the 34th ACM SIGPLAN Conference on Programming Language Design and Implementation. p. 423–434. PLDI ’13, Association for Computing Machinery, New York, NY, USA (2013). 10.1145/2491956.2462191, 10.1145/2491956.2462191

[14] Li, Y., Tan, T., Møller, A., Smaragdakis, Y.: A principled approach to selective context sensitivity for pointer analysis. ACM Trans. Program. Lang. Syst. **42**(2) (May 2020). 10.1145/3381915, 10.1145/3381915

[15] Li, Y., Tan, T., Sui, Y., Xue, J.: Self-inferencing reflection resolution for java. In: Jones, R. (ed.) ECOOP 2014 - Object-Oriented Programming. pp. 27–53. Springer Berlin Heidelberg, Berlin, Heidelberg (2014), 10.1007/978-3-662-44202-9_2

[16] Li, Y., Tan, T., Xue, J.: Effective soundness-guided reflection analysis. In: Blazy, S., Jensen, T. (eds.) Static Analysis. pp. 162–180. Springer Berlin Heidelberg, Berlin, Heidelberg (2015), 10.1007/978-3-662-48288-9_10

[17] Li, Y., Tan, T., Xue, J.: Understanding and analyzing java reflection. ACM Trans. Softw. Eng. Methodol. **28**(2) (Feb 2019). 10.1145/3295739, 10.1145/3295739

[18] Liu, J., Li, Y., Tan, T., Xue, J.: Reflection analysis for java: Uncovering more reflective targets precisely. In: 2017 IEEE 28th International Symposium on Software Reliability Engineering (ISSRE). pp. 12–23 (2017), 10.1109/ISSRE.2017.36

[19] Milanova, A., Rountev, A., Ryder, B.G.: Parameterized object sensitivity forpoints-to analysis for java. ACM Trans. Softw. Eng. Methodol. **14**(1), 1–41 (Jan 2005). 10.1145/1044834.1044835, 10.1145/1044834.1044835

[20] Ramalingam, G.: The undecidability of aliasing. ACM Trans. Program. Lang. Syst. **16**(5), 1467–1471 (Sep 1994). 10.1145/186025.186041, 10.1145/186025.186041

[21] Reif, M., Kübler, F., Eichberg, M., Helm, D., Mezini, M.: Judge: Identifying, Understanding, and Evaluating Sources of Unsoundness in Call Graphs. In: Proceedings of the 28th ACM SIGSOFT International Symposium on Software Testing and Analysis (to appear). ISSTA 2019 (2019). 10.1145/3293882.3330555, 10.1145/3293882.3330555

[22] Scholz, B., Jordan, H., Subotić, P., Westmann, T.: On fast large-scale program analysis in datalog. In: Proceedings of the 25th International Conference on Compiler Construction. pp. 196–206. CC 2016, ACM, New York, NY, USA (2016). 10.1145/2892208.2892226, 10.1145/2892208.2892226

[23] Sharir, M., Pnueli, A.: Two approaches to interprocedural data flow analysis. New York Univ. Comput. Sci. Dept., New York, NY (1978), https://cds.cern.ch/record/120118

[24] Smaragdakis, Y., Balatsouras, G.: Pointer analysis. Found. Trends Program. Lang. **2**(1), 1–69 ( 2015). 10.1561/2500000014, 10.1561/2500000014

[25] Smaragdakis, Y., Balatsouras, G., Kastrinis, G., Bravenboer, M.: More sound static handling of java reflection. In: Feng, X., Park, S. (eds.) Programming Languages and Systems - 13th Asian Symposium, APLAS 2015, Pohang, South Korea, November 30 - December 2, 2015, Proceedings. Lecture Notes in Computer Science, vol. 9458, pp. 485–503. Springer (2015). 10.1007/978-3-319-26529-2_26, 10.1007/978-3-319-26529-2_26

[26] Smaragdakis, Y., Bravenboer, M., Lhoták, O.: Pick your contexts well: Understanding object-sensitivity. In: Proceedings of the 38th Annual ACM SIGPLAN-SIGACT Symposium on Principles of Programming Languages. pp. 17–30. POPL ’11, ACM, New York, NY, USA (2011). 10.1145/1926385.1926390, 10.1145/1926385.1926390

[27] Smaragdakis, Y., Kastrinis, G.: Defensive Points-To Analysis: Effective Soundness via Laziness. In: Millstein, T. (ed.) 32nd European Conference on Object-Oriented Programming (ECOOP 2018). Leibniz International Proceedings in Informatics (LIPIcs), vol. 109, pp. 23:1–23:28. Schloss Dagstuhl-Leibniz-Zentrum fuer Informatik, Dagstuhl, Germany (2018). 10.4230/LIPIcs.ECOOP.2018.23, http://drops.dagstuhl.de/opus/volltexte/2018/9228

[28] Soot: Soot - a framework for analyzing and transforming java and android applications (Jan 2019), http://sable.github.io/soot/

[29] Späth, J., Ali, K., Bodden, E.: Ideal: Efficient and precise alias-aware dataflow analysis. In: 2017 International Conference on Object-Oriented Programming, Languages and Applications (OOPSLA/SPLASH). ACM Press (Oct 2017), 10.1145/3133923

[30] Späth, J., Ali, K., Bodden, E.: Context-, flow-, and field-sensitive data-flow analysis using synchronized pushdown systems. Proc. ACM Program. Lang. **3**(POPL), 48:1–48:29 (2019). 10.1145/3290361, 10.1145/3290361

[31] Späth, J., Do, L.N.Q., Ali, K., Bodden, E.: Boomerang: Demand-driven flow- and context-sensitive pointer analysis for java. In: Krishnamurthi, S., Lerner, B.S. (eds.) 30th European Conference on Object-Oriented Programming, ECOOP 2016, July 18-22, 2016, Rome, Italy. LIPIcs, vol. 56, pp. 22:1–22:26. Schloss Dagstuhl - Leibniz-Zentrum für Informatik (2016). 10.4230/LIPIcs.ECOOP.2016.22, 10.4230/LIPIcs.ECOOP.2016.22

[32] Sui, L., Dietrich, J., Emery, M., Rasheed, S., Tahir, A.: On the soundness of call graph construction in the presence of dynamic language features - a benchmark and tool evaluation. In: Ryu, S. (ed.) Programming Languages and Systems. pp. 69–88. Springer International Publishing, Cham (2018), 10.1007/978-3-030-02768-1_4

[33] Sui, L., Dietrich, J., Tahir, A., Fourtounis, G.: On the recall of static call graph construction in practice. In: Proceedings of the ACM/IEEE 42nd International Conference on Software Engineering. p. 1049–1060. ICSE ’20, Association for Computing Machinery, New York, NY, USA (2020). 10.1145/3377811.3380441, 10.1145/3377811.3380441

[34] Tan, T., Li, Y., Xue, J.: Efficient and precise points-to analysis: Modeling the heap by merging equivalent automata. In: Proceedings of the 38th ACM SIGPLAN Conference on Programming Language Design and Implementation. pp. 278–291. PLDI 2017, Association for Computing Machinery, New York, NY, USA (2017). 10.1145/3062341.3062360

[35] Vallée-Rai, R., Co, P., Gagnon, E., Hendren, L., Lam, P., Sundaresan, V.: Soot - a java bytecode optimization framework. In: Proceedings of the 1999 Conference of the Centre for Advanced Studies on Collaborative Research. p. 13. CASCON ’99, IBM Press (1999), 10.5555/781995.782008

[36] Vallée-Rai, R., Gagnon, E., Hendren, L., Lam, P., Pominville, P., Sundaresan, V.: Optimizing java bytecode using the soot framework: Is it feasible? In: Watt, D.A. (ed.) Compiler Construction. pp. 18–34. Springer Berlin Heidelberg, Berlin, Heidelberg (2000), 10.1007/3-540-46423-9_2

[37] WALA: Watson libraries for program analysis (Jan 2019), http://wala.sourceforge.net/wiki/index.php/Main_Page

[38] Wala: Intermediate representation (IR) (Aug 2020), https://github.com/wala/WALA/wiki/Intermediate-Representation-(IR)

[39] Wala: Pointer analysis (Aug 2020), https://github.com/wala/WALA/wiki/Pointer-Analysis

[40] Wei, F., Roy, S., Ou, X., Robby: Amandroid: A precise and general inter-component data flow analysis framework for security vetting of android apps. ACM Trans. Priv. Secur. **21**(3) (Apr 2018). 10.1145/3183575, 10.1145/3183575

[41] Wikipedia: Datalog (Jan 2019), https://en.wikipedia.org/wiki/Datalog

